# In the details: the micro-ethics of negotiations and in-situ judgements in participatory design with marginalised children

**DOI:** 10.1080/15710882.2020.1722174

**Published:** 2020-02-13

**Authors:** Katta Spiel, Emeline Brulé, Christopher Frauenberger, Gilles Bailley, Geraldine Fitzpatrick

**Affiliations:** a HCI Group, TU Wien, Vienna University of Technology, Vienna, Austria; b eMedia Research Lab, KU Leuven, Leuven, Belgium; c Department of Engineering and Design, University of Sussex, Brighton, UK; d ISIR Laboratory, Sorbonne Université, CNRS, Paris, France

**Keywords:** Children, marginalisation, participatory design, ethics

## Abstract

Engaging marginalised children, such as disabled children, in Participatory Design (PD) entails particular challenges. The processes can effect social changes by decidedly attending to their lived experience as expertise. However, involving marginalised children in research also requires maintaining a delicate balance between ensuring their right to participation as well as their protection from harm. The resulting tensions are politically charged, affected by myriads of power differences and create moral dilemmas. We present seven case studies, drawing from two participatory design research projects. They illustrate the in-situ judgements taken to address specific dilemmas and provide nuanced insights into the trade-offs required by child-led participatory design processes. Subsequently, we identify three challenges: positioning our work to the children’s carers’ values, protecting ourselves, and enabling the (relative) risk-taking associated with participation for children. We call for this micro-ethical approach to be used when reporting research ethics in practice, and as a guidance for the training of researchers and practitioners.

## 1. Introduction

Involving children in research, particularly in participatory research, comes with heightened ethical challenges (Holland et al. 2010). Children belonging to marginalised groups, such as disabled children,^1^ are particularly vulnerable, hence requiring additional considerations around notions of power and responsibilities. These considerations require us to challenge core ethical principles and assumptions as their ways of consenting, assenting or dissenting to research are fundamentally different compared to engaging with adult populations (Tisdall, Kay, and Punch 2012; Antle 2017; McNally et al. 2016). However, there is broad consensus that involving such groups is essential, both regarding processes and outcomes (Mankoff, Hayes, and Kasnitz 2010). It avoids grounding the design of technologies on the assumptions of developers and de-signers about the lived experience of their target group, which, in turn, could further contribute to their overall marginalisation (Robertson and Wagner 2012). However, engaging with marginalised children in design processes challenges designers to more directly think about how they position themselves and their work relative to these principles (Brulé and Spiel 2019).Hence, caring about ethics generally and how to enact reflexivity in-situ, is, fundamentally, a moral and political question tied to the transformative aspirations of participatory design research (Pihkala and Karasti 2016). Children’s prerogative to participate in decisions affecting their lives is established by the United Nations (UN) Convention on the Rights of the Child. Consequently, it is critical to establish ethical frameworks and guidelines for such research collaborations, as well as foster a culture of continuous discussion and reflection to improve practices (Graham et al. 2013).

The majority of guidelines aims to establish generic best-practices, with respect to researcher’s behaviour or research design. They may also underline the importance of the researcher’s virtue and caring skill (Graham et al. 2013). These ethical principles are established prior to the research, anticipating known and expected risks, and expressed as preparatory checklists to go through. However, while conducting activities with marginalised children, researchers are required to make situated judgements on the spot, which are often politically charged – particularly as these pertain to questions of power – and may be unforeseeable or, in consequence, create a contradiction to the over-arching ethical principles of participation and protection (Frauenberger, Rauhala, and Fitzpatrick 2016).

We argue that such situated judgements, which often remain tacit and implicit, need to be explicitly foregrounded and examined. Hence, we suggest extending the notions of ethics of researchers-participants collaboration in participatory design (PD) (Robertson and Wagner 2012) with the notion of using *micro-ethics* in participatory design with marginalised children. Understanding PD through micro-ethics means looking at the ethically charged small in-situ decisions that shape the relationship as well as the resulting design work. Here, we expand on our previous work (Spiel et al. 2018) by refocusing our work on how to design with heterogeneous groups of children.

After reviewing fundamental concepts in ethics and related work in participatory design (PD) with marginalised children, we examine seven case studies of participatory design with groups of disabled children which illustrate micro-ethical issues and decisions. We unpack our motivations, impacts, and weigh alternative decisions we could have taken. Some challenges reoccurred across both projects: positioning our work to the children’s carers’ values, protecting ourselves, and enabling the (relative) risk-taking associated with participation for children. Finally, we articulate an under- standing of micro-ethics in the context of PD with the view to speak to researchers and practitioners alike.

## 2. Background

We argue that the main reference frame for participatory design – bar a few examples (e.g. Pihkala and Karasti (2016)) – rely on anticipatory ethics. In contrast, our research approach, in part due to a lack of institutional ethics boards, is rooted in care ethics (Tronto 2001).To ensure ethical research conduct, many institutions require researchers to have their research designs reviewed before starting the research. However, institutionalised ethics focus primarily on deontology while *enacted* research leads to challenges better addressed at a micro-level. Our approach to micro-ethics, hence, complements prefigurative strands of ethical considerations.

### 2.1. Research ethics

#### 2.1.1. Theory: on normative and applied ethics

Ethics is concerned with the study of morality, e.g. what constitutes a good life and, consequently, how we should live (Deigh 2010). Within this field, *normative research ethics* focus on determining what general laws should be followed in research, and *applied ethics* look into how we can ethically reflect on specific issues. Our research projects combined a set of standard deontological guidelines (e.g. informed consent) with a strong focus on researchers’ virtue, which is consistent with recommendations for research with children (Graham et al. 2013; Alderson and Morrow 2011) and with the roots of virtue ethics in participatory design (Steen 2011). In addition, we specifically reflected on their ability to *care. Care ethics* postulate that all beings are interdependent. The approach highlights the often under-valued relations of care. Care supposes:

(1) being attentive to others’ needs, (2) taking responsibility for responding to them, (3) being skilled in providing care, while (4) being mindful of the potential abuses of care (both for the care-giver and the care-receiver (Barnes et al. 2015) and the subjective perspectives of others on the care received (Tronto 2001). Care ethics is also a political theory: it bears consequences for political institutions and policy development (Popke 2003). A care-based approach cannot be separated from social, community objectives – such as, in the case of participatory design, the transformative agenda to support both democratic involvement and individual’s self-determination (Morris 2001).

#### 2.1.2. Application: institutional ethical approval

Institutional ethical approval of research, i.e. Internal Review Boards, often focuses on ensuring that deontological rules such as autonomy, beneficence, nonmaleficence, and justice are respected. Individually, these boards come with different requirements at each institution (Larson et al. 2004). In both our case studies, there was no institutional review available, leaving the ethically sound conduct of said research in our hands.^2^ Such an absence poses a risk but it also provides an opportunity for researchers to more carefully reflect on their practices.

#### 2.1.3. Micro-ethics

Initially developed for health care contexts, *micro-ethics* focus on seemingly mundane, yet ethically charged matters: the presentation of food in hospitals, the language used by doctors etc. – ‘what happens in every interaction’ between individuals (Komesaroff 1995). The concept is also dialectic: decisions made by the patient also have ethical significance. For Komesaroff (1995), prescriptive ethical principles are ineffective, as they are subject to change in situations to which they could be applied: there can only be themes and practical cases. In the field of engineering and computing, ‘“micro-ethics” include concern with individuals and the internal relations of the engineering profession’ (Herkert 2005). The focus is on professional codes of conduct, such as being attentive to the risks embedded in a system’s design (Bittner and Hornecker 2005), and contrasts these codes with taking responsibilities for the larger societal impact of a technology.

Participatory Design (PD) research is historically concerned with a transformative agenda inbuilding systems with and for people (Ehn and Badham 2002). PD researchers care about providing participants with opportunities to express their creativity (Steen 2011). A PD researcher would be careful about turn-taking in discussion, for instance, because of these guidelines. We propose a micro-ethical approach closer to that of Komesaroff (1995) and focus on *relationships between researchers and marginalised children* as they unfold. We further argue ethics are produced through ‘doing ethics’ or rather making situated judgements that feed back into a larger understanding of what ethical conduct means for a society (Lynch 2016). Focusing on micro-ethical decisions requires researchers to explicitly reflect on the values underlying their actions, how they understand participants’ values, and the dialectic production of their relationship.

### 2.2. Ethics in participatory design with marginalised children

#### 2.2.1. Ethical principles and approaches

Historically, participatory design aimed at reinforcing democracy by acknowledging and supporting a diversity of perspectives (Halskov and Hansen 2015) – often framed as inherently attentive to ethics and caring relationships (Steen 2011). Robertson and Wagner (2012) identified four central ethical questions for PD researchers: (1) Who do we engage as participants? (2) How do we engage with them? (3) How do we represent them? (4) What can we offer in return?

To answer (one or several of) these questions, some scholars develop or apply ethical theories to participatory design (Gram-Hansen and Ryberg 2016). Others propose practical ethics frameworks and guidelines (Van Mechelen et al. 2014; Read et al. 2013). Further work discusses the researchers themselves, and how their assumptions shape the values emerging in the research process and its outcomes (Malinverni and Pares 2017; Pihkala and Karasti 2016). Finally, a few have investigated the often implied beneficial long-term effects of participatory design – and found them limited (Ehn and Badham 2002; Carsten Stahl 2014).

The limitations of exclusively anticipatory and principles-based ethics have been discussed in a series of workshops at HCI (Human-Computer Interaction) venues. Key researchers have argued that the increasingly explorative, contextual and value-driven nature of engagements requires a more nuanced approach, which they call *situational ethics* (Waycott et al. 2015, 2016; Munteanu et al. 2015; Malinverni and Pares 2017). Frauenberger, Rauhala, and Fitzpatrick (2016) make a similar argument for complementing anticipatory ethics approaches by a reflective design practice guided by collectively negotiated moral principles. With reference to the concept of a reflective practitioner by Schön (1987), they call this *in-action ethics*.

However, most research on moral decisions in HCI research has focused on how to reach consensus between the different actors or trying to explain how this affects specific design projects. In complementary contrast, our approach highlights unresolved disagreements on what constitutes the right course of action and acknowledges the impossibilities of reaching consensus in certain situations.

#### 2.2.2. Ethics of participatory design with children

Research with children also holds participation as a principle, allowing us to overcome ethical challenges particularly pertaining children’s agency (Thomas and Kane 1998; Graham et al. 2013). Yet, participation does not per se have the expected impact. Scholarship on participatory design with children acknowledges power differentials between adults and children as well as limits of participation. Areas of concern specific to children include making sure they are fully aware of what their participation entails, finding specific ways to report to children and adequate means of participation. Researchers have come up with checklists (Read et al. 2013) and recommendations (McNally et al. 2016) to address potential risks and ensure the children’s protection.

#### 2.2.3. The case of marginalised children

What qualifies as marginalisation can be vastly different, including the experience of seeking asylum, being disabled, living in a low-income household, growing up with adoptive or foster parents, being fat or a person of colour. While not an exhaustive list, it illustrates the diversity through which marginalisation can occur – often in more than one aspect (Schlesinger, Edwards, and Grinter 2017; Erete, Israni, and Dillahunt 2018).

The experiences of marginalised children are often neglected in research and policies. We focus on marginalised children as a way to counterbalance this under-representation (Watson 2012), which is consistent with the aims of participatory design. However, some scholars have argued that focusing on marginalisation may backfire and essentialise inequalities and that marginalised groups may oppose such categorisation (Morris 2016; Nygreen 2006; Watson 2002).Specifically attending to marginalised people has the potential to do both: enable resistance and reinforce biases against marginalised groups; the very first non-resolvable ethically charged tension.

Even when researchers establish a relationship with children that aims at minimising the power differences between them, multiple ethical aspects play into any participatory research. Researchers are older than children and their statements are given more validity within society. Marginalised children are often not directly attended to, and their accounts are continuously (mis-)interpreted and re-framed in publications and research reports. As researchers, we need to be aware of these experienced power differences and how they actively shape collaborations; we need to be especially careful to monitor who is making which decisions (Bratteteig and Wagner 2012).

Especially in longer-term collaborations, children build up trust towards the researchers. It may result in researchers becoming aware of private and confidential information, which is of less concern the other way around. Hence, careful management of hierarchies and how they might be subverted in the interest of the children becomes paramount (Abebe 2009). In particular, we want to emphasise that ‘children may exploit, appropriate, redirect, contest or refuse participatory techniques’ (Gallagher 2008). Such subversive strategies of the children can be identified and then encouraged – especially with marginalised children as they are often limited in expressing resistance they can exercise in their daily life.

To account for these tensions in participatory design with marginalised children, our work focuses on situated moral *judgements* made by researchers when working with marginalised children. The originality of this paper resides in the micro-scale we use to look at ethics, and the groups we worked with.

## 3. Case studies

We conducted two participatory design projects, *Social Play Technologies* and *MapSense*, with disabled children. In Social Play Technologies we engaged with groups of disabled children, allistic and autistic, whereas, in MapSense, we collaborated with visually impaired children. The projects were conducted by two different research groups. In both cases, the children faced systemic adversarial attitudes by society and, occasionally, their peers due to their atypical behaviour and communication modes. Through understanding the children as marginalised, we frame disability as a physical difference as well as a social exclusion (Morris 1991), hence creating a lens through which we position the children with agency over their own life (Spiel et al. 2019).

### 3.1. Social Play Technologies

In the Social Play Technologies^3^ project, two of the authors co-designed (together with others) technologies facilitating social play with groups consisting of four to six autistic and allistic children. At the time of writing, we worked with three groups creating individually suitable technologies that aid the children in realising their visions for play. Previously, we reported on the agonistic qualities of design processes with neurodiverse groups of children (Frauenberger et al. 2019).

Autism is diagnosed along a triad of characteristics (American Psychiatric Association 2000). Autistic people experience difficulties with neurotypical modes of communication, interaction and imagination. We follow an understanding of autism as a variation on a neurodiverse spectrum (Singer 1999).

While the institution in which Social Play Technologies was carried out did not require any formal ethics approval, we were guided by a collective ethos that was partly articulated, partly implied. We recorded this in an extensive document which acknowledged potential issues and benefits for parents, teachers, children and researchers as well as strategies to resolve anticipated tensions between stakeholders. The formalised ethics in these documents framed the research activities. However, we noticed how we had to make several ethical judgements emerging in-situ and for which the consequences were not always clear. These are the types of ethical encounters, we describe.

#### 3.1.1. Attending to differing needs

**Figure 1.**
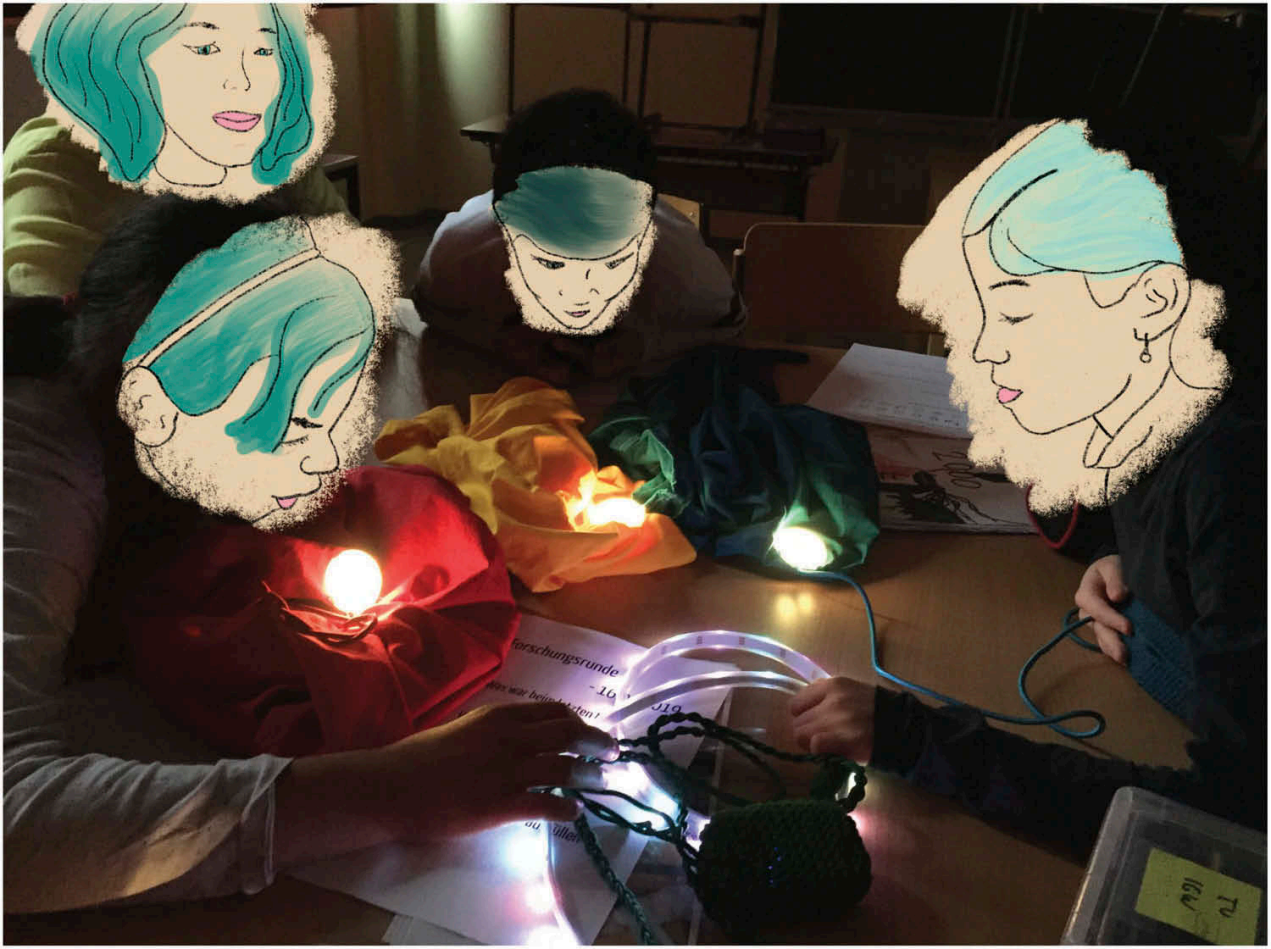
Interacting with one prototypes within social play technologies, one child coming at it with stern curiosity, one with perky interest and another one calmly investigating.

In the context of our groups, different neurodivergent conditions were present across and within children, making these needs often more pressing given the neurotypically oriented expectations of the environment. Researchers have to allocate their attention between a general and a specific perspective, always having an overview of individual needs and how they can be negotiated on a group level (see Figure 1). However, attention is also a limited resource particularly in design contexts, where the rigid norms the children are used to from educational contexts are not always desired or helpful.

Group 1 consisted of four children, one of which was diagnosed with autism. One of the neurotypically presenting children, Ian, was very creative, immersed in the narratives we offered, and full of ideas he constantly wanted to share. While this is excellent for design endeavours, it leads to issues if one researcher is entirely caught up by attending to Ian while the remaining children need to share the attentional resources of only one other person. In such cases, our initial judgement was to let the attention

follow the child as this comprised a successful strategy in a previous group. However, in this case, we needed to adjust and find different ways of allocating attention as to not frustrate the remaining three children.

These situations require assessments, judgements and multiple corrections in rapid successions. For such a process to succeed, researchers need to be able to identify potentially problematic situations quickly and explicitly reflect on their role in-action.

#### 3.1.2. Attuning the self

Group dynamics within any group of children are highly volatile and can quickly change the context of design. It is challenging to identify the points of where strict boundaries have to be enforced to enable the overall Handlungsspielraum (taken from Makhaeva, Frauenberger, and Spiel 2016) for all children present. This is particularly the case for design work that requires letting go of controlling the context of the children and their actions to allow for creative expressions (De Jaegher 2019). Even if researchers are judicious about how they allocate their attention, the result might not always be optimal and, occasionally, impact the productivity of a design session.

In another group, one child had difficulty managing their emotions due to early childhood trauma. Quin quickly reacted to situations and were often very upset. In many cases, we could anticipate these moments and divert Quin’s attention to other aspects of the design process or reframe their experiences in a more positive light. In one case though, a prototype did not exhibit the desired stability for Quin to realise their ideas. Additionally, other group members tried to interact with the technology with their own intentions without communicating them. We did not understand early enough that this was very upsetting to Quin and failed to point them to productive strategies before they started actively and violently destroying parts of the technological setup. As a reaction, one of the researchers quickly indicated to the others present that one adult would be solely paying attention to Quin now to resolve the situation. They also switched from a playful and collegial approach to a more serious and strict tone that explicitly set boundaries and identified the destruction of the prototype as a point of contention while also making space for Quin to articulate their perspective, needs and desires. In rejoining the group, they reframed the situation positively as an opportunity to thoroughly test the robustness of the material.

**Figure 2.**
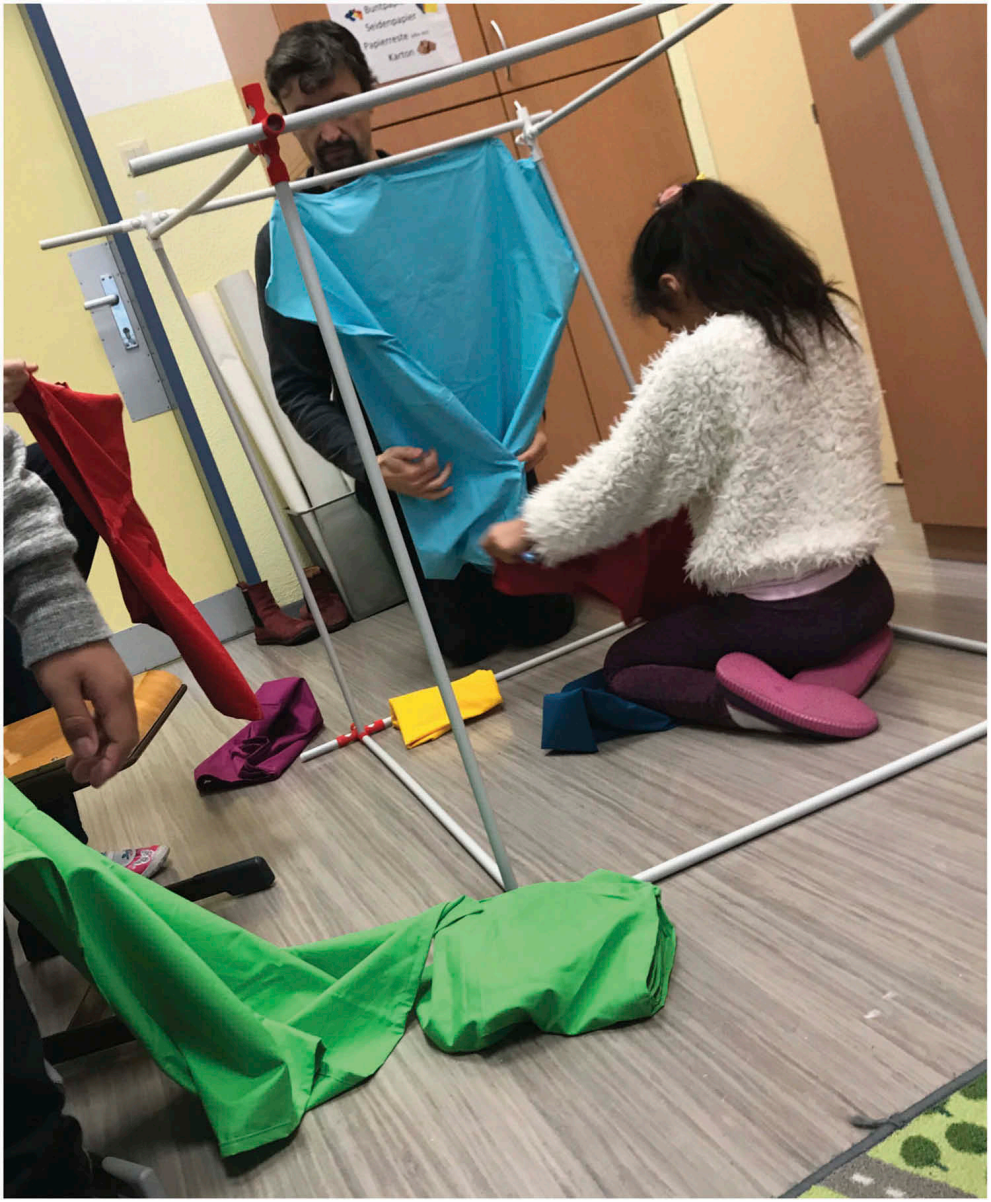
Creative engagements on the object with one design research attending to a specific child, some creating independently and the other design researcher quickly shifting between taking a picture and tentatively attending to the group.

Being prepared for free collaborative play with participants while acknowledging a responsibility of care that includes setting safe boundaries for everyone involved, are just two potential poles of available research roles. These should be coordinated so that participating children can find a suitable partner for their needs and desires (potentially ranging from structured engagement or free expressiveness) even if one of the researchers has to take on a more defined role on one of the ends of this spectrum (see also, Figure 2). However, even if researchers will be required to resolve conflict situations that are destructive to the design process, they need to acknowledge their limitations in resolving underlying issues for their participants.

#### 3.1.3. Acknowledging outsider role

When working with groups of children, pre-existing relationships often come with already established power dynamics. The relationships between the children are further developed within but also, more importantly, outside of the design sessions with researchers. As such, researchers will always remain outsiders to these dynamics. However, the children’s relationships, conflicts and communal understanding seep into design, which means these are fundamental to consider for PD researchers.

Within a third group of children, Xenia, who had been diagnosed with Trisomy 21, was often excluded from playing with the other four children (two with an Autism diagnosis, one with an ADHD diagnosis). She was furthermore the only child growing up without speaking Turkish in the home. Hence, we assume that the reasons for this exclusion cannot easily be pinpointed towards single characteristics but are, instead, *intersectional* in nature (Crenshaw 1990). Often, she also pre-emptively took herself out of group activities or seemed to deliberately disrupt them. As we were only present for an hour every other week, we framed the design activities as a shared experience that everyone could contribute to in their preferred modes of engagement; however, this was easily overridden by the already existing group dynamics.

Hence, while it can be desirable to nudge group dynamics and hierarchies into alternative directions particularly when it comes to design, researchers need to acknowledge their position as outsiders. Their reach is limited in that they engage with the children only in short episodes and for a fixed amount of time. In many cases, no infinite process or involvement will be possible to work on these structures long term. Being aware of this outsider position helps with assessing the potential of overall impact appropriately. However, it also allows researchers to set explicit rules within the design sessions that acknowledge existing dynamics but also dispel them for the context of the sessions.

### 3.2. Mapsense

In MapSense, we co-designed technologies with visually impaired children and their teachers or therapists (Brulé and Jouffrais 2016; Brulé et al. 2016).We explored the design of technologies for more enjoyable experiences in the classroom for children in primary and secondary school while supporting adults’ educational goals (Brulé 2018). In initial interviews, the children had often framed the classroom as an adversarial space. Rather than validating the usability or educational gains of prototypes, we studied how a design process might modify the relationships between children and teachers. During this two-year ethnographic study, we designed more than ten different probes and prototypes, used by 15 children (Brulé and Bailly 2018; Brulé et al. 2018).

The institutional frame for MapSense did not have a formalised process for ethical review. Thus, we drew on the UNICEF’s guidelines for ethics in research with children (Graham et al. 2013), and literature on care ethics in action research (Williamson and Prosser 2002). Furthermore, we kept a detailed auto-ethnographic diary for continuous reflection (similarly to Malinverni and Pares 2017). While we had conducted a substantial literature review for structural, ethical issues (e.g. how to handle differentials of power between adults and children participants), there were situations that required rapid ethical decisions with unclear consequences at the time.

#### 3.2.1. Preserving relationships with adult gatekeepers

**Figure 3.**
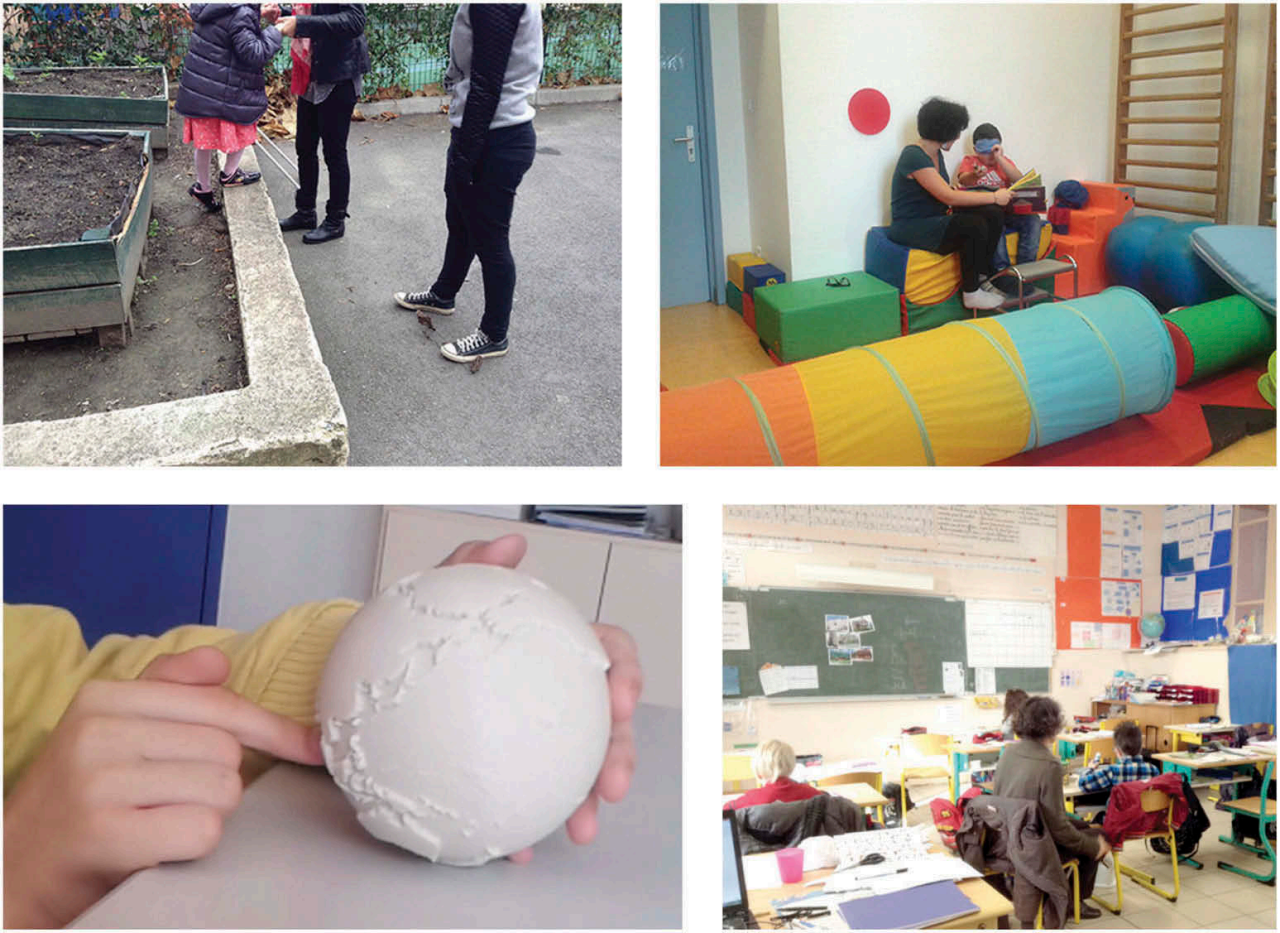
In the Mapsense project, design activities were embedded within the normal delivery of educational services. These pictures illustrate various challenges encountered: the top right and left picture show how close physically to the pupils educators and teachers need to be in different activities. The same might go for the designer. Bottom left picture is of the tactile globe probe which proved challenging to deploy as educators used it as a teaching opportunity that ultimately caused the pupil to disengage. Finally, the bottom right photo depicts a classroom context, in which a special education teacher sits by one of our participant, further illustrating issues of negotiating with adult gatekeepers. The case study on confidentiality is not illustrated, due to its very nature.

We tried to not constrain the children in their ways of participating to the project. However, this was not the case for the established adults taking care of them (hereby named ‘carers’). For instance, a carer wanted to observe a child manipulating a probe (a 3D printed tactile globe, see Figure 3). While the researcher was interested in the child’s comments and critiques, asking only questions for clarification, the carer wanted to transform this activity into a formal learning task. She started asking restrictive questions or making comments such as ‘no, you’re wrong’. To not compromise our collaboration with the carers, which was fundamental for collaborating with the children, we retreated from the interaction by slightly moving away and remaining silent. After a few minutes, the child stopped answering and turned away. Such withdrawal from interaction occurred several times: when we tried to help a child regulating their emotions, his teacher intervened and told the child to cry somewhere else. In another case, a carer wanted a teenager to demonstrate how to use newly adapted computer software but the teenager refused.

In these interactions, we had to balance two versions of trust: maintaining the trust of child participants *and* maintaining the trust of carers to be able to work further with children (which in the carers’ views includes ‘*enforcing discipline*’, and *‘making children do what they’re supposed to do’*). Additionally, we were concerned about preserving the conditions for the participatory design research. For instance, we communicated that there were no right or wrong answers in a design process. However, withdrawing from interaction also meant that we did not directly engage carers on the subject of educational norms, even though this might have been beneficial for the children as well as the carers. Furthermore, we let children go through an apparently distressing experience without intervention from our side, which affects the trust in the researcher.

#### 3.2.2. Demonstrations of affection

Younger children, or children with multiple impairments, often initiated physical contact. Such interactions involved hugging, haptically exploring the body of the researcher (which can be part of how visually impaired children get to know and engage with people), and activities such as dancing. We did not refuse any of the contacts initiated by the children, as we considered this a form of relationship building. We also found that these activities were an essential part of building trust. However, we sometimes felt uncomfortable, were unsure of how children understood our relationship and how we could manage it. To protect both, children and researchers, there were always at least two or three adult researchers present.

At stake was the interchange of care: whereas it is accepted for researchers to care for children, it feels dangerous to take care of children given that researchers’ presence is temporary. Furthermore, discussing the children’s expectations was difficult, as this was not something they were used to doing. Children would sometimes forget about previous meetings, while at other times they would not. Judging the appropriateness of this form of engagement and its consequences was thus difficult to foresee. This raises the question of how we develop rapport with participants, who do not have a shared mode in which to manage and reflect on social relationships.

Our strategy was to adopt a transparent mode of communication, based on the acknowledgement of the exchanges with them and the duration of the study. We informed them every time when we would come back and when we would ultimately leave and re-assured them that the probes and prototypes would stay with them. Indeed, these were framed as gifts, or proofs of interest and affection by the children. However, adequate distancing in research relationships with the children remains an open question. It is similarly unclear to what extent we can ask researchers to actively become comfortable with physical interactions.

#### 3.2.3. Confidentiality

In a different instance, during a research interview, a 17-year-old male-presenting teenager asked questions about the romantic life of the (25-year-old) field researcher. When she indicated that such questions were inappropriate, he propositioned her further. The organisation staff had described this teenager as ‘*deviant*’.

The researcher reminded him that he was a minor and that she was acting in a formalised working setting. She also explained that declining to answer his first questions marked the refusal of such interactions, a refusal that should be respected. She encouraged him to ask questions about romantic relationships to a trusted adult and informed him of an official website, which provides sex education resources and contacts with educators and social workers to teenagers. He replied that this was just something he often attempted with young (female) adults he met. The researcher immediately stopped the interview after this. She then purposefully decreased the frequency of interactions and the incident remained isolated.

Three ethical issues are at stake: First, the researcher was not trained to discuss these topics with a teenager. Second, even though the resources provided were designed by a governmental agency, making this call can be opposed by other carers. Third, if repeated, we would have to disclose this behaviour for our own comfort even though we guaranteed confidentiality. In-situ, we provided him with external resources and, not wishing to fuel discourses labelling him as deviant, we did not report the incident.

## 4. Discussion

Across all case studies, we identified three challenges, each a moral principle we were committed to: (1) adopting a pluralistic view of ethics, in line with care ethics and participatory design (PD) ideals, which entailed positioning our work to children’s carers’ values and agendas as well, (2) protecting participants as well as ourselves, and (3) enabling (relative) risk-taking associated with participation for children, required for child-led PD processes. We now expand on these themes, as a starting point for an understanding of micro-ethics in participatory design with marginalised children.

### 4.1. Negotiating multiple values and agendas

As social workers, medical personnel, specialised teachers, therapists or family members, carers set the structure of children’s life. In our case studies, carers framed the research in unexpected ways. Our strategies in negotiating with them were manifold, all of them with unforeseeable consequences at the point where they had to be enacted.

- We withdrew when a co-researcher or other adult acted contrary to our judgement and only discussed our concerns afterwards. While this meant that children were potentially exposed to negative experiences, this approach seemed more effective in a setting where we would not threaten a carer’s authority as a by-product. As the carers were gatekeepers to the participatory research, we had to partly adhere to their desires – even if that meant acting contrary to desirable conditions for participatory *design* work. This inherently complicates virtue ethics proclaiming child-led processes.
- We established alternative approaches to working with the children. In part, this meant excluding or ignoring parts of their context during the design process for the benefit of opening up new spaces in which empowerment and design activities were possible. Together with the withdrawing strategy, however, this potential space becomes fleeting and insecure as it can only happens through precariously balancing the values of carers and researchers alike.
- We carefully navigated the influence of different carers and mediated on topics with pre-existing tensions between carers and children. We would either not bring up a controversial issue or change the topic with reference to external sources.

These strategies show that even in participatory design with child-led processes, carers play a relevant role. As such, their tangential role in participatory design with marginalised children might be under-conceptualised. In our cases, their presence made us carefully prioritise certain topics over others, which meant they had circumstantial influence on the participatory design work, its outcomes and/or use contexts for the resulting prototypes. Hence, an analysis of Carer-Children-Researcher relationships through an ethical lens could additionally increase an understanding of how our processes are shaped. That way, caring strategies in research can be negotiated explicitly – appropriate to the level of involvement.

### 4.2. Protecting participants and ourselves

When working with marginalised children, risks become at the same time more explicit *and* more implicit. We have to take care of the marginalised children and ensure that we are not exposing them to harm, but we are also vulnerable ourselves. When children are invited to long-term research collaborations needed for participatory design, they are also enabled to build complex personal relationships with the design researchers. While this might allow them to make new experiences and widen their horizons, this is based on developing personal relationships increasing the vulnerability of children and PD researchers alike.

How to appropriately balance professional conduct and personal relationships is a matter that can only be practically engaged with; anticipatory deliberations remain theoretical and speculative (Lynch 2016). In the case where a participant displayed inappropriate conduct during a research activity, we had to weigh the risk of the child being impacted disproportionately in the future, the risk of the researcher who was unsure how to appropriately handle the situation and who was in a position of liability, and the adherence to the confidentiality of the meeting which has been ensured to participants before they engaged in the research.

Hence, participatory design projects not only pose potential risks to children but also to researchers. These risks are not always physical, but might also affect mental health, the career of people involved, the development of the children and, ultimately, also the design process and its outcomes. While researchers might not ever be able to eliminate or foresee all risks, it helps to be aware of their potential and consider which choices might lead to which potential outcomes and the attached risks for researchers and children or other stakeholders in the participatory processes alike. As a core point of care ethics, all participants in the research – researchers as well as marginalised children – are vulnerable and ‘at-risk’ when they collaborate (Tronto 2001). It is, ultimately, a matter of our judgement to limit the risks for people, in a context where the most appropriate procedure is not necessarily clear-cut.

It matters who embodies the research. Different bodies invite different modes of interaction. Children engaged with the uncommon bodies of researchers in curious, playful and exploratory ways. Through the comparatively long collaborations, the engagement with the children built closer relationships. Our work with marginalised children gave us additional insights into the importance of positioning ourselves transparently as researchers towards the children and their social environment. Professional and personal aspects played into the participatory design research (see also, Brulé and Spiel 2019).

### 4.3. Enabling participation

In both our projects, we envisioned the processes as child-led. We understood the children as design partners who were not only shaping but leading the design. In practice, however, we had to carefully negotiate with the children about their level of participation – particularly in contexts where adult attention is a limited resource.

Another aspect of caring for the children required us to be mindful of the responsibility put on us as researchers not only by the adult environment but by the children themselves. They trusted us to keep them safe. Such trust is fundamental for a productive participatory design relationship that requires mutual vulnerability to enable processes in which creativity can be dared. At the same time, we created a space in which they should also feel free to express themselves individually and explore the boundaries of what they know (including rules). As researchers, we tried to engage with the children at eye-level in a relationship of equal partners. Yet, situations may occur where researchers need to assert authority to keep everyone safe. In negotiating different needs of marginalised children, the design process and the researchers themselves, we need to find a balance between rejecting and embracing responsibility, between equal partnership and care, between creative openness and ensuring safety.

## 5. Micro-ethics for participatory design with marginalised children

We argued for the necessity of reflection on *micro-ethics:* judgements that cannot always be foreseen. They may not seem immediately relevant but offer rich insights into the participatory design process. Hence, it becomes all the more important to be *reflective* design practitioners (Schön 1987). We propose an understanding of micro-ethics for participatory design research with marginalised children and offer a few suggestions on how to actualise them in-situ.

### 5.1. Micro-ethics for participatory design

Micro-ethics provide a lens to look into the seemingly mundane everyday activities that contribute to ethical conduct on a larger scale (Komesaroff 1995). The approach echoes the concern of other scholars to report more systematically on situated moral decisions and how they affect research relationships and design outcomes (e.g., Malinverni and Pares 2017).We started by identifying the range of moral principles and perspectives involved in the participatory design project. We then used this framework to analyse difficult moral decisions in-situ and outlined alternative decisions we could have taken, given the overarching ethical principles. Finally, we delineated expected consequences for alternative choices. This process of systematically linking situated ethical decisions to the ethical frameworks and range of moral perspectives comprises the main contribution our work offers.

In Table 1, we list the three challenges discussed in the previous section, together with the micro-ethical strategies we employed encountering similar situations. It illustrates the space of negotiations between different stakeholders. Taking into account the full context in which children live, required us to make contestable prioritisations. Protecting children while enabling participation can only be evaluated as a dynamic in which potentials and risks have to be carefully considered, especially in a group. Child-led participatory design (PD) requires researchers to negotiate between all associated stakeholders; an endeavour that requires sensitivity, nuance and, at least to some extent, political finesse.

**Table 1.** Tensions between ethical principles stemming from virtue ethics and strategies used in micro-ethics.

Ethical Principles	Strategies in Micro-Ethics
Negotiating contradictory values and agendas	Navigation of carers priorisation of topics making judgements
Child-led PD: enabling participation	Negotiation of needs being responsible complex risk assessment
Protecting ourselves	Personal relationships commitment to participants embodied research

Micro-ethics is an invitation to focus on areas of dissent, and on relationships within and surrounding PD processes – also as a resource for design. It requires careful navigation of values that were not initially in focus. For instance, emphasising *child-led* PD might obfuscate our responsibilities as adults to both protect from harm and encourage the kind of risk-taking that offers positive experiences. Or the values we hold and embody, that shape our professional conduct, sometimes need to be tweaked to accommodate a child’s holistic context.

While these tensions are ever-present in participatory design, they remain underreported, even though the relationships between all involved participants bear potentially tremendous implications on the design work. However, such impacts might be difficult to quantify or evaluate. Instead of ignoring these tensions, we suggest critically engaging with them to better understand how ethical principles are enacted in-situ, at a micro-level, and how they affect participatory design as a discipline and practice.

### 5.2. Practical suggestions

We suggest identifying ethically charged situations after each encounter with participants, determining the choices and the judgements made and then reflecting on them with others, preferably people who are not directly involved in the design work since shared assumptions within a group might hinder the reflective practice. Methods used in research on ethics are similarly suitable for this reflection; including, for instance, analysing the same situation using different ethical theories, basing it on different moral values and principles, or discussing preferred choices.

With marginalised children, researchers have to pay close attention to the children’s abilities and preferences concerning the high cognitive and sometimes even physical demands of participatory design work. To avoid overwhelming participants, it might be appropriate to partly include additional children with different characteristics. For example, Ruland, Starren, and Vatne (2008) conducted participatory design with children with cancer and used groups of children without cancer at some points in the design process to negotiate between different needs. While this might leave marginalised children out of parts of the design process, which consequently leads to them not having direct influence over those parts, such an approach can be deemed appropriate in some cases. Agency, participation and what is possible to ask for without ‘tyrannising’ (Cooke and Kothari 2001) the children has to be continually conceptualised anew for each PD collaboration and, ultimately, each encounter with marginalised children. We argue that researchers have to be especially careful when aiming for child-led design processes with marginalised children as, for example, younger children might not have the same vocabulary or skills to express their ideas and desires as the researchers. Researchers have to be attuned to explicitly making space for the participation of the children on their own terms (Spiel et al. 2017; Williams and Gilbert 2019).

The vulnerability of marginalised children made these ethical difficulties more salient. However, the disagreements, or the difficulties to identify a ‘good’ choice, are likely to be present in all PD contexts. Encouraging the publication the use of micro-ethics to report on participatory design processes and challenges, might open new perspectives and provide the field with more insights into how these choices affect outcomes. This could also be beneficial for the training of researchers and practitioners. Case studies are very often used in ethics classes in other fields, which could be taken up by HCI and PD. Furthermore, examination of micro-ethics might make them more attuned to kindness and care (Dweck 2017). In the situations in which researchers have to make ethical judgements, they often cannot know or assess beforehand whether a decision was right, correct or even just the best available, particularly given the intricacy of multiple ethical strands in the research. Often, it is our task to *judge when different choices are available*. Without making excuses, we then need to be kind towards ourselves and others, reflect on those choices and discuss them, learn from them and improve our capabilities to make ethically sound judgements in the moment, also to improve the basis of our design-related knowledge.

## 6. Conclusion

We illustrated a range of micro-ethical decisions, how and when they are made, and their implications for research relationships in participatory design with marginalised children. We argue that, as a community, we should be aware that in engaging in participatory design we continuously make similar judgements and decisions. Such an awareness comprises not only a personal choice in terms of reflection, but also a political necessity given the transformative character of participatory design work. Though we focus here on marginalised children, we emphasise that most of the examples could potentially occur in any participatory research setting. One limitation of this work is that it is based on personal experiences of the key researchers only, instead of emerging from a shared account between researchers, research teams, children participants and their adult gatekeepers.

Future work in this area would benefit from an even deeper theoretical integration to the field of ethics. In particular, we envision exciting parallels with Haraway’s recent work on ethics advocating for ‘*staying with the trouble*’, which refer to unresolvable ethical concerns (Haraway 2016). Our work can also be expanded in the field of design education and training, as ethically guided practice, for now and in our own experience, is something that each researcher needs to figure out along the way, without dedicated external guidelines to reflect on.

Our contribution is, hence, twofold: the first is pragmatic and resides in the empirical grounding of complex judgements during interactions with marginalised children in participatory research. The second is the articulation of an approach to ethics which combines normative ethics frameworks and situated moral judgements made over the course of the research through the analytic lens of micro-ethics.

## Notes

1. Following arguments made in Disability Studies (e.g. Sinclair 2013), we use identity-first language as to not *other* disabled people by the use of a language construct specifically designed for them.

2. This remains relatively common in some European contexts to this day, notably France and Austria, where our research was conducted.

3. http://www.socialplay.at.
